# Co-colonization of methicillin-resistant *Staphylococcus aureus* and *Candida* spp. in children with malignancies

**DOI:** 10.1186/s13568-024-01667-7

**Published:** 2024-02-13

**Authors:** Raziyeh Baharvand, Fatemeh Fallah, Parvaneh Jafari, Leila Azimi

**Affiliations:** 1 Department of Microbiology, Faculty of Science, Islamic Azad University, Arak branch, Arak, Iran; 2 Pediatric Infections Research Center, Research Institute for Children’s Health, Shahid Beheshti University of Medical Sciences, Tehran, Iran

**Keywords:** *Staphylococcus aureus*, Methicillin-resistant *Staphylococcus aureus*, *Candida* Spp., Children, Malignancy, Iran

## Abstract

This study aimed to evaluate the interaction between methicillin-resistant *Staphylococcus aureus***(**MRSA) and *Candida* spp. in the oral cavity of children with malignancies under chemotherapy. We evaluated the expression level of *Als3p* and *mecA* in *Candida* spp. and MRSA strains in both single colonization and co-colonization condition. Oral and nasal samples were collected by dry sponge swabs in 10 ml of sterile phosphate-buffered saline. The MRSA and *Candida* spp. was confirmed using the PCR method and *mecA* and *Als3p* genes, respectively. The SYBR Green-based quantitative real-time PCR was used to evaluate the relative expression levels of *mecA* and *Als3p* genes in MRSA and *Candida* spp., respectively. The frequency of *S. aureus* in oral-only and nasal-only swab samples were 14.1% (*n* = 24/170). 58.3% (*n* = 14/24) and 29.2% (*n* = 7/24) of *S. aureus* isolated from oral and nasal samples were MRSA, respectively. Among *Candida* species, *C. albicans* (*n* = 28/170; 16.5%) had the highest frequency. The oral co-colonization of MRSA and *Candida* spp. was detected in 4.7% (*n* = 8/170) patients. The overall average of gene expression levels among all *Candida* spp. and MRSA isolates indicated that the *mecA* and *Als3p* genes expression increased six and two times in co-colonization conditions compared to single colonization conditions, respectively. Our findings revealed the importance of polymicrobial infection in clinical settings and stated that it is possible that *Candida* spp. facilitates the infection of *S. aureus* and can lead to systemic infection in co-colonized patients.

## Introduction

Methicillin-resistant *Staphylococcus aureus* (MRSA) and *Candida* species, especially *C. albicans* are two of the most dangerous microorganisms that cause serious hospital-acquired infections(Wu et al. 2017; Nasser et al. 2022). Although the different pathogens can create severe infections in humans, *Staphylococcus aureus* (*S. aureus*) and *C. albicans* are ranked as the second and third bloodstream pathogens causing higher rates of mortality in hospitalized individuals(Zago et al. 2015). These two pathogens are isolated in mucosal surfaces and wound infections(Schlecht et al. 2015). *S. aureus* has several virulence factors such as fibronectin-binding proteins A and B, chemotaxis inhibitory protein of staphylococci, and alpha-toxin, which allows the microorganism to attach to the host cells, evade the host immune response, and induces tissue damage in host cells, respectively(Todd and Peters 2019, Nasser et al. 2020). Among *S. aureus*, MRSA is associated with multidrug resistance and typically leads to higher levels of mortality(Chen, Huang and Infection 2014, Noshak et al. 2023). It is predicted that MRSA is responsible for about 52% of severe nosocomial infections such as systematic blood infection and aspiration pneumonia among patients hospitalized in intensive care units(NNIS 1999, Terpenning et al. 2001, Mylotte 2002). Similar to *S. aureus*, *Candida* species are considered significant healthcare-associated microorganisms related to several systemic and non-systemic infections(Lin et al. 2013). *C. albicans* is the most predominant and infectious of the *Candida* species(Martins, Koga-Ito and Jorge 2002). *C. albicans* have several virulence factors including the production of toxins such as candidalysin, polymorphism and morphological switching from the rounded yeast to the invasive hyphal form, ability to biofilm formation, invasins such as agglutinin-like sequence 3 adhesin (Als3p), and metabolic adaptation, which allows the *Candida* to cause systemic diseases(Sheehan et al. 2020). The mortality rate of *C. albicans* in systemic infections is approximately 50%(Schlecht et al. 2015). Thirty until 60% of infections caused by *C. albicans* are polymicrobial infections and among bacterial pathogens, *S. aureus* is the most frequently isolated pathogen from these infections(Rodrigues, Gomes and Rodrigues 2020). In general, *S. aureus* and *C. albicans* are found in the oral cavity and are human oral microbiota(Bassis et al. 2014). However, in patients with immunodeficiency disorders, these opportunistic microorganisms can cause an array of infections including urinary tract infection, periodontitis, ventilator-associated pneumonia, denture stomatitis, and keratitis, which are typically difficult to treat(Pate, Jones and Wilhelmus 2006, Shariati et al. 2020). It is acknowledged that *S. aureus* can adhere specifically to the invasive hyphal elements (Als3p) in the *Candida* cell wall and disseminate in host cells and finally invade host tissues(Schlecht et al. 2015; Zago et al. 2015). Moreover, it is suggested that modulation of virulence factors expression such as *Als3p* in *Candida* and *mecA* in *S. aureus* accelerates systemic staphylococcal infection(Sheehan et al. 2020). Therefore, *Als3p* is the main *Candida* target for *S. aureus* binding(Wu et al. 2017). According to what has been said, the knowledge regarding the co-colonization of *Candida* species and *S. aureus* especially MRSA strains in children with malignancies under chemotherapy is critical. However, little data is available about the frequency of these pathogens among these patients. The present study aimed to evaluate the frequency of MRSA and *Candida* spp. in the oral cavity of children with malignancies under chemotherapy. Moreover, we evaluate the expression level of *Als3p* and *mecA* genes in *Candida* species and MRSA strains in both single colonization and co-colonization condition.

## Materials and methods

### Study population and sampling

This cross-sectional, single-center study enrolled patients with different malignancies under chemotherapy at Shahid Beheshti Medical University Hospital, Mofid hospital from January 2018 to October 2020. Mofid children’s hospital is one of the most popular children’s hospitals in Tehran, the capital of Iran. The aims of the study had been clarified to included children and samples taken from them after written informed consent was signed by parents. Patients whose parents did not sign the consent form were excluded from the study. After informed consent, clinical data collected included demographic characteristics such as sex, age, weight, clinical and laboratory information such as a history of surgery, cancer, chemotherapy, acute or chronic kidney disease, neutropenia, diabetes, having a central venuse catheter, having a foley catheter, having an active wound on the body, abdominal pain, history of organ transplantation, history of antifungal and antibacterial consumption, and the type of disease, underlying malignancy, and history of hospitalization in the intensive care units (ICUs). Oral and nasal samples were collected by dry sponge swabs in 10 ml of sterile phosphate-buffered saline (PBS, 0.1 M, pH 7.2) and transported to the microbiology laboratory at the pediatric infectious research center, Shahid Beheshti University of Medical Sciences, Tehran, Iran.

### Bacterial and fungal isolates and species identification

To test for the presence of *S. aureus*, the oral and nasal swabs were inoculated into specific media within 4 h of arrival. The bacterial culture media were as follows: Mannitol salt agar, sheep blood agar, and CHROMagarTM Staph aureus (Sa) (CHROMagarR Company, Paris, France. Conventional tests such as catalase, coagulase, DNase tests, and growth on 6% NaCl were performed on Gram-positive cocci after the Gram staining method. According to the Clinical and Laboratory Standards Institute (CLSI) criteria(Humphries et al. 2021), the resistance to oxacillin among *S. aureus* isolates (MRSA) was determined using the Kirby Bauer Disk Diffusion method. *S. aureus* ATCC 33,591 and ATCC 29,213 were used as methicillin-resistant and methicillin sensitive control strains, respectively.

For Candida species identification, all swab samples were cultured on the Sabaouraud Dextrose agar containing chloramphenicol, CHROM agar candida medium (Merck, Germany), germ tube formation in fetal calf serum at 37 °C, cornmeal agar with Tween*-*80, and direct microscopic observation(Ardehali et al. 2019).

### DNA extraction and polymerase chain reaction (PCR)

The final identification of MRSA isolates was performed using the PCR method by a specific *mecA* gene. For this purpose, the genomic DNA of *S. aureus* isolates was extracted using a DNA extraction kit (GeNet Bio Company, Daejeon, Korea; Cat. No, K-3000) and frozen at -80 °C until use. The primer used for the PCR reaction is shown in Table 1. The amplification reaction was performed in 35 cycles on a thermal cycler (Eppendorf, Mastercycler Gradient; Eppendorf, Hamburg, Germany) and the PCR program of *mecA* is followed by initial denaturation (One cycle of 95 °C for 5 min), denaturation (45 s at 94 ^˚^C), annealing (45 s at 60 ^˚^C), elongation (30 s at 72 ^˚^C), and final elongation step (5 min at 72 ^˚^C). PCR material volumes have been described previously by Shariati et al(Shariati et al. 2020).

**Table 1:** Primers used for amplification of genes

Primers name		Sequence (5’→3’)
*mecA*	F	TCCAGATTACAACTTCACCAGG
	R	CCACTTCATATCTTGTAAGG
16s rRNA	F	CAGCTCGTGTCGTGAGATGT
	R	CGTAAGGGCCATGATGACTT
*Als3*	F	CAACTTGGGTTATTGAAACAAAAACA
	R	AGAAACAGAAACCCAAGAACAACCT
*Its*	F	TCCTCCGCTTATTGATATGC
	R	TCCGTAGGTGAACCTGCGG

Genomic DNA extraction of *Candida* spp. colonies were carried out using the specific kit based on the manufacturer’s instructions and frozen at -80 °C. The final identification of *Candida* spp. was performed using a PCR assay based on a specific *Als3p* gene. The primer used for the PCR reaction is shown in Table 1. PCR material volume and amplification condition have been described previously by Ardehali et al(Ardehali et al. 2019). Finally, PCR products were stained by DNA-safe stain (SinaClon Co., Iran) and screened by gel electrophoresis on 1.5% agarose gel under UV light.

### Semiquantitative RT-PCR

According to the manufacturer’s instructions, an RNeasy Mini Kit (SinaClon) was applied to the total RNA extraction of *S. aureus* and *Candida* species. In the next step, all extracted RNA was treated with DNase I (Fermentas, Waltham, MA, USA) and then were suspended in 50 µl of diethylpyrocarbonate (DEPC) treated water (0.1% v/v). Nanodrop (DS-11 Spectrophotometer, USA) was used to determine the total RNA concentration. Moreover, we used from Takara kit (Shiga, Japan) for the complementary DNA synthesis, and the reaction conditions have been described by Ardehali et al(Ardehali et al. 2019).

The SYBR Green-based quantitative real-time PCR (qRT-PCR) was used to evaluate the relative expression levels of *mecA* and *Als3p* genes MRSA and *Candida* species, respectively. The primers used for qRT-PCR are shown in Table 1. The qRT-PCR reaction was performed on a Rotor-Gene RT-PCR machine (Corbett Research, Sydney, Australia; model RG3000, software version 6). RT-PCR conditions have been described previously by Zhang et al(Zhang et al. 2016). We used from 16 S ribosomal RNA housekeeping gene to normalize of relative expression of *mecA* and *Als3p* genes and the results were analyzed based on the 2^−ΔΔCt^ method. All data were included in the statistical package SPSS v.23.0 (SPSS Inc., Chicago, IL, USA) and were analyzed using Chi-Square test. A *P*-value of < 0.05 was set as significant.

### GenBank submission ID numbers

GenBank submission ID numbers for the two surveyed genes were as follows:

1. *mecA*: 2631311
2. *Als3p*: 2633263

## Results

### Study population

In the present study, from January 2018 to October 2020, 170 children who have different malignancies were included consecutively and were surveyed for nasal and oral *S. aureus* and *Candida* spp. colonization in Mofid children’s hospital. The clinical characteristics and demographical information of all patients are shown in Tables 2 and 3. In general, 102 (60%) and 68 (40%) nasal and oral swab samples were collected from males and females, respectively. Among the all patients, 38.8% (*n* = 66/170) and 31.8% (*n* = 54/170) were in the age range of 1 to 5 years and 6–10 years, respectively. The weight of 43.5% (*n* = 74/170) of all patients ranged from 11 to 20 kg (Kg). The duration of hospitalization in 40.6% (*n* = 69/170) of patients was 4 to 7 days. Based on the self-declaration, 76.5% (*n* = 130/170) and 39.4% (*n* = 67/170) of patients have a history of surgery and ICU admission, respectively. Among all patients, Acute lymphocytic leukemia (ALL), blood cancers, and dialysis with 14.7% (*n* = 25/170), 10% (*n* = 17/170), and 8.8% (*n* = 15/170) frequency were main reasons for hospitalization. All dialysis patients were oncological. Moreover, 55.9% of all patients had a record of antibiotic therapy. On the other hand, out of 170 patients included in the current study, only 62 (36.5%) made use of antifungal drugs including voriconazole, fluconazole, and nystatin.

**Table 2:** Baseline characteristics of patients and frequency of *S. aureus* and *Candida* spp. in oral samples

Characteristics	Total (%)	S. aureus	*p*-value	MRSA	*p*-value	Candida spp.	*p*-value
Gender							
Male	102 (60)	16 (15.7)	0.472	9 (8.8)	0.733	22 (21.6)	0.878
Female	68 (40)	8 (11.8)		5 (7.3)		14 (20.6)	
Age							
<=1	15 (8.8%)	3 (20)/ 0 (0)	0.711	1 (6.7)	0.700	4 (26.7)	0.796
1–5	66 (38.8)	11 (16.7)		6 (9)		13 (19.7)	
6–10	54 (31.8)	6 (11.1)		4 (7.4)		10 (18.5)	
11–15	29 (17.1)	3 (10.3)		2 (6.9)		8 (27.6)	
16–20	3 (1.8)	1 (33.3)		1 (33.3)		0 (0)	
> 20	3 (1.8)	0 (0)		0 (0)		1 (33.3)	
Weight							
0–5	2 (1.2)	1 (50)	0.320	1 (50)	0.341	0 (0)	0.584
6–10	24 (14.1)	6 (25)		2 (8.3)		5 (20.8)	
11–20	74 (43.5)	9 (12.2)		7 (9.4)		14 (18.9)	
21–30	32 (18.8)	4 (12.5)		3 (9.4)		8 (25)	
31–40	18 (10.6)	3 (16.7)		1 (5.5)		2 (11.1)	
41–50	7 (4.1)	1 (14.3)		0 (0)		2 (28.6)	
> 50	13 (7.6)	0 (0)		0 (0)		5 (38.5)	
Duration of hospitalization							
0–3	60 (35.3)	11 (18.3)	0.130	6 (10)	0.938	16 (26.7)	0.453
4–7	69 (40.6)	9 (13)		6 (8.7)		10 (14.5)	
8–10	20 (11.8)	0 (0)		1 (5)		6 (30)	
11–15	13 (7.6)	4 (30.7)		1 (7.7)		3 (23)	
16–20	3 (1.8)	0 (0)		0 (0)		0 (0)	
< 20	5 (2.9)	0 (0)		0 (0)		1 (20)	
Surgery history							
Yes	130 (76.5)	18 (13.8)	0.855	11 (8.5)	0.847	27 (20.8)	0.815
No	40 (23.5)	6 (15)		3 (7.5)		9 (22.5)	
History of ICU admission							
Yes	67 (39.4)	12 (17.9)	0.252	8 (11.9)	0.156	20 (29.9)	0.026
No	103 (60.6)	12 (11.7)		6 (5.8)		16 (15.5)	
Chemotherapy							
Yes	148 (87.1)	18 (12.2)	0.058	10 (6.7)	0.069	32 (21.6)	0.713
No	22 (12.9)	6 (27.3)		4 (18.2)		4 (18.2)	
Cancer							
Yes	146 (85.9)	18 (12.3)	0.099	9 (6.2)	0.015	31 (21.2)	0.965
No	24 (14.1)	6 (25)		5 (20.8)		5 (20.8)	
Renal failure							
Yes	34 (20)	5 (14.7)	0.912	2 (5.9)	0.577	2 (5.9)	0.015
No	136 (80)	19 (14)		12 (8.8)		34 (25)	
Central venous catheter							
Yes	35 (20.6)	8 (22.8)	0.096	0 (0)	0.004	4 (11.4)	0.113
No	135 (79.4)	16 (11.8)		14 (10.4)		32 (23.7)	
Foley catheter							
Yes	7 (4.1)	1 (14.3)	0.990	7 (100)	0.418	1 (14.3)	0.649
No	163 (95.9)	23 (14.1)		7 (4.3)		35 (21.5)	
Neutropenia							
Yes	28 (16.5)	5 (17.8)	0.534	3 (10.7)	0.602	5 (17.8)	0.638
No	142 (83.5)	19 (13.4)		11 (7.7)		31 (21.8)	
Organ transplantation							
Yes	20 (11.8)	5 (25)	0.137	4 (20)	0.042	3 (15)	0.472
No	150 (88.2)	19 (12.7)		10 (6.7)		33 (22)	

**Table 3:** Baseline characteristics of patients and frequency of *S. aureus* and *Candida* spp. in nasal samples

Characteristics	Total (%)	S. aureus	*p*-value	MRSA	*p*-value	Candida spp.	*p*-value
Gender							
Male	102 (60)	19 (18.6)	0.039	6 (5.9)	0.156	3 (2.9)	1
Female	68 (40)	5 (7.3)		1 (1.5)		2 (2.9)	
Age							
<=1	15 (8.8%)	0 (0)	0.058	0 (0)	0.153	0 (0)	0.062
1–5	66 (38.8)	8 (12.1)		0 (0)		2 (3)	
6–10	54 (31.8)	8 (14.8)		4 (7.4)		1 (1.8)	
11–15	29 (17.1)	5 (17.2)		3 (10.3)		1 (3.4)	
16–20	3 (1.8)	2 (66.7)		0 (0)		0 (0)	
> 20	3 (1.8)	1 (33.3)		0 (0)		1 (33.3)	
Weight							
0–5	2 (1.2)	0 (0)	0.067	0 (0)	0.076	0 (0)	0.006
6–10	24 (14.1)	2 (8.3)		1 (4.2)		1 (4.2)	
11–20	74 (43.5)	8 (10.8)		0 (0)		2 (2.7)	
21–30	32 (18.8)	8 (25)		4 (12.5)		0 (0)	
31–40	18 (10.6)	3 (16.7)		1 (5.5)		0 (0)	
41–50	7 (4.1)	3 (42.8)		1 (14.3)		2 (28.6)	
> 50	13 (7.6)	0 (0)		0 (0)		0 (0)	
Duration of hospitalization							
0–3	60 (35.3)	11 (18.3)	0.824	4 (6.7)	0.292	1 (1.7)	0.747
4–7	69 (40.6)	8 (11.6)		1 (1.4)		3 (4.3)	
8–10	20 (11.8)	2 (10)		1 (5)		0 (0)	
11–15	13 (7.6)	2 (15.4)		0 (0)		1 (7.7)	
16–20	3 (1.8)	0 (0)		0 (0)		0 (0)	
< 20	5 (2.9)	1 (20)		1 (20)		0 (0)	
Surgery history							
Yes	130 (76.5)	21 (16.1)	0.169	7 (5.4)	0.134	4 (3)	0.850
No	40 (23.5)	3 (7.5)		0 (0)		1 (2.5)	
History of ICU admission							
Yes	67 (39.4)	8 (11.9)	0.511	1 (1.5)	0.165	3 (4.5)	0.339
No	103 (60.6)	16 (15.5)		6 (5.8)		2 (1.9)	
Chemotherapy							
Yes	148 (87.1)	16 (10.8)	0.001	4 (2.7)	0.016	4 (2.7)	0.633
No	22 (12.9)	8 (36.4)		3 (13.6)		1 (4.5)	
Cancer							
Yes	146 (85.9)	16 (10.9)	0.004	4 (2.7)	0.026	4 (2.7)	0.701
No	24 (14.1)	8 (33.3)		3 (12.5)		1 (4.2)	
Renal failure							
Yes	34 (20)	9 (26.5)	0.021	3 (8.8)	0.123	1 (2.9)	1
No	136 (80)	15 (11)		4 (2.9)		4 (2.9)	
Central venous catheter							
Yes	35 (20.6)	9 (25.7)	0.027	3 (8.6)	0.137	1 (2.8)	0.974
No	135 (79.4)	15 (11.1)		4 (3)		4 (3)	
Foley catheter							
Yes	7 (4.1)	2 (28.6)	0.262	1 (14.3)	0.167	0 (0)	0.638
No	163 (95.9)	22 (13.5)		6 (3.7)		5 (3)	
Neutropenia							
Yes	28 (16.5)	5 (17.8)	0.534	1 (3.6)	0.874	0 (0)	0.314
No	142 (83.5)	19 (13.4)		6 (4.2)		5 (3.5)	
Organ transplantation							
Yes	20 (11.8)	4 (20)	0.421	1 (5)	0.833	1 (5)	0.562
No	150 (88.2)	20 (13.3)		6 (4)		4 (2.7)	

**Prevalence of*S. aureus*and MRSA colonization**.

As shown in Tables 2 and 3, the frequency of *S. aureus* in oral-only and nasal-only swab samples was 14.1% (*n* = 24/170). Oral-only and nasal-only colonization by MRSA was found in 8.2% (*n* = 14/170), and 4.1% (*n* = 7/170) patients, respectively. 58.3% (*n* = 14/24) and 29.2% (*n* = 7/24) of *S. aureus* isolated from oral and nasal samples were MRSA, respectively. Among all patients, both oral and nasal colonization by *S. aureus* was found in 5.3% (*n* = 9/170) patients.

*S. aureus* had the highest frequency among nasal samples collected from males than females (18.6% versus 7.3%; *p* = 0.039). The frequency of MRSA isolates was more among nasal samples collected from males. However, these differences were not statistically significant (*p* = 0.156).

Interestingly, there was an inverse relationship between having a history of cancer and central venous catheter with the frequency of MRSA isolates in oral samples of patients. Patients without a history of cancer and who do have not a central venous catheter showed the highest frequency of MRSA in oral samples (*p* = 0.015 and *p* = 0.004). In patients with a history of organ transplantation, oral colonization by MRSA was high (*p* = 0.042).

The nasal colonization of *S. aureus* in patients with central venous catheters and renal failure was high (*p* = 0.027 and *p* = 0.021).

The correlation between antibiotics usage and the frequency of *S. aureus* and MRSA in oral and nasal samples are shown in Tables 4 and 5. Among patients with antibiotic (Cotrimoxazole) therapy, oral and nasal colonization with *S. aureus* was low (2.8%; *p* = 0.032 and 0%; *p* = 0.007, respectively).

**Table 4:** Correlation between antibiotics usage with the frequency of *S. aureus*, MRSA, and *Candida* spp. in oral samples

Antibiotics	Total (%)	S. aureus	*p*-value	MRSA	*p*-value	Candida spp.	*p*-value
Antibiotic history							
Yes	95 (55.9)	14 (14.7)	0.794	9 (9.5)	0.509	25 (26.3)	0.065
No	75 (44.1)	10 (13.3)		5 (6.7)		11 (4.7)	
Ciprofloxacin							
Yes	19 (11.2)	4 (21)	0.357	3 (15.8)	0.204	3 (15.8)	0.542
No	151 (88.2)	20 (13.2)		11 (7.3)		33 (21.8)	
Vancomycin							
Yes	19 (11.2)	3 (15.8)	0.824	3 (15.8)	0.204	9 (47.4)	0.003
No	151 (88.2)	21 (13.9)		11 (7.3)		27 (17.9)	
Cotrimoxazole							
Yes	35 (20.6)	1 (2.8)	0.032	1 (2.8)	0.194	3 (8.6)	0.041
No	135 (79.4)	23 (17)		13 (9.6)		33 (24.4)	
Amikacin							
Yes	10 (5.9)	2 (20)	0.582	1 (10)	0.834	3 (30)	0.481
No	160 (94.1)	22 (13.7)		13 (8.1)		33 (20.6)	
Metronidazole							
Yes	10 (5.9)	0 (0)	0.186	0 (0)	0.329	1	0.373
No	160 (94.1)	24 (15)		14 (8.7)		35 (21.9)	
**Antifungal history**							
Yes	62 (36.5)	8 (12.9)	0.730	5 (8)	0.951	15 (24.2)	0.466
No	108 (63.5)	16 (14.8)		9 (8.3)		21 (19.4)	
Voriconazole							
Yes	8 (4.7)	2 (25)	0.365	1 (12.5)	0.653	1 (12.5)	0.538
No	162 (95.3)	22 (13.6)		13 (8)		35 (21.6)	
Fluconazole							
Yes	13 (7.6)	2 (15.4)	0.891	1 (7.7)	0.941	1 (7.7)	0.216
No	157 (92.4)	22 (14)		13 (8.3)		35 (22.3)	
Nystatin							
Yes	28 (16.5)	2 (7.1)	0.246	2 (7.1)	0.818	6 (21.4)	0.972
No	142 (83.5)	22 (15.5)		12 (8.4)		30 (21.1)	

**Table 5:** Correlation between antibiotics usage with the frequency of *S. aureus*, MRSA, and *Candida* spp. in nasal samples

Antibiotics	Total (%)	S. aureus	*p*-value	MRSA	*p*-value	Candida spp.	*p*-value
Antibiotic history							
Yes	95 (55.9)	10 (10.5)	0.130	3 (3.1)	0.478	4 (4.2)	0.270
No	75 (44.1)	14 (18.7)		4 (5.3)		1 (1.3)	
Ciprofloxacin							
Yes	19 (11.2)	5 (26.3)	0.105	2 (10.5)	0.136	0 (0)	0.421
No	151 (88.2)	19 (12.6)		5 (3.3)		5 (3.3)	
Vancomycin							
Yes	19 (11.2)	2 (10.5)	0.633	1 (5.3)	0.790	0 (0)	0.421
No	151 (88.2)	22 (14.6)		6 (4)		5 (3.3)	
Cotrimoxazole							
Yes	35 (20.6)	0 (0)	0.007	1 ((2.8)	0.674	0 (0)	0.248
No	135 (79.4)	24 (17.8)		6 (4.4)		5 (3.7)	
Amikacin							
Yes	10 (5.9)	0 (0)	0.186	0 (0)	0.499	0 (0)	0.570
No	160 (94.1)	24 (15)		7 (4.4)		5 (3.1)	
Metronidazole							
Yes	10 (5.9)	0 (0)	0.186	0 (0)	0.499	0 (0)	0.570
No	160 (94.1)	24 (15)		7 (4.3)		5 (3.1)	
**Antifungal history**							
Yes	62 (36.5)	7 (11.3)	0.422	3 (4.8)	0.720	1 (1.6)	0.437
No	108 (63.5)	17 (15.7)		4 (3.7)		4 (3.7)	
Voriconazole							
Yes	8 (4.7)	1 (12.5)	0.893	0 (0)	0.548	0 (0)	0.614
No	162 (95.3)	23 (14.2)		7 (4.3)		5 (3)	
Fluconazole							
Yes	13 (7.6)	1 (7.7)	0.489	0 (0)	0.437	0 (0)	0.514
No	157 (92.4)	23 (14.6)		7 (4.4)		5 (3.2)	
Nystatin							
Yes	28 (16.5)	1 (3.6)	0.080	0 (0)	0.230	0 (0)	0.314
No	142 (83.5)	23 (16.2)		7 (4.9)		5 (35.2)	

### **Prevalence of*Candida*colonization**

The frequency of *Candida* spp. among included patients is shown in Tables 2 and 3. Among the 170 patients, oral colonization by *Candida* species was found in 36 (21.2%) patients, while nasal colonization by candida species was found in 5 (2.9%) patients. Based on the conventional phenotypic methods, the frequency of *Candida* species was as follows: *C. albicans* (*n* = 28/170; 16.5%), *C. glabrata* (*n* = 10/170; 5.9%), *C. tropicalis* (*n* = 2/170; 1.2%), and *C. krusei* (*n* = 0/170; 0%).

*Candida* spp. had the highest frequency in oral samples collected from patients who have a history of ICU admission (29.9%; *p* = 0.026). The oral colonization of *Candida* spp. among patients with renal failure was low (*p* = 0.015).

The frequency of *Candida* spp. among patients with antibacterial and antifungal therapy is shown in Tables 4 and 5. Results showed that oral colonization of *Candida* spp. among patients with vancomycin therapy was high (47.4%; *p* = 0.003). In contrast, our analyses revealed that oral colonization of *Candida* spp. among patients with cotrimoxazole therapy was low (8.6%; *p* = 0.041).

Results showed that oral and nasal colonization of *Candida* spp. among patients with antifungal therapy (voriconazole and fluconazole) was low (12.5% versus 21.6% and 7.7% versus 22.3%). However, these differences were not statistically significant (*p* = 0.538 and *p* = 0.216).

### **Co-colonization of MRSA and*Candida*spp.**

The oral and nasal co-colonization of the *S. aureus* and *Candida* spp. was detected in 5.9% (*n* = 10/170) and 0.6% (*n* = 1/170) patients, respectively. Moreover, the oral co-colonization of MRSA and *Candida* spp. was detected in 4.7% (*n* = 8/170) patients. We could not detect nasal co-colonization between MRSA and *Candida* spp.

### **The expression levels of*mecA*and*Als3p*genes**

The expression levels of *mecA* and *Als3p* genes were compared among 10 MRSA and 10 *Candida* spp. in single and co-colonization conditions. Quantitative analysis indicated that *Als3p* gene expression increased from two times to more than four times in co-colonization conditions compared to single colonization conditions. On the other hand, our analyses revealed that among MRSA isolates, the *mecA* gene expression increased from four times to more than seven times in co-colonization conditions compared to single colonization conditions. The overall average of gene expression levels among all *Candida* spp. and MRSA isolates indicated that the *mecA* and *Als3p* genes expression increased six times and two in co-colonization conditions compared to single colonization conditions, respectively.

## Discussion

In general, polymicrobial infections have resistance to the majority of antimicrobials, and treatment of these infections is difficult(Eichelberger and Cassat 2021). In most cases, polymicrobial infections are associated with aggressive forms of diseases(Todd 2021). *S. aureus* and *Candida* spp. are the main opportunistic microorganisms isolated from human infections(Kean et al. 2017). Globally, both microorganisms are commensals colonizing human mucosal surfaces and are the leading opportunist pathogens causing hospital- and community-acquired infections(Carolus et al. 2019). Although the knowledge regarding the co-colonization of *Candida* species and *S. aureus* in children with malignancies under chemotherapy is critical, however, little data is available about the frequency of these pathogens among these patients, worldwide.

In the present study, we surveyed the frequency of *S. aureus*, MRSA, and *Candida* species in both single colonization and co-colonization conditions in the oral cavity and nasal of children with malignancies under chemotherapy. Moreover, we evaluated the expression level of *Als3p* and *mecA* genes in *Candida* spp. and MRSA strains in both single colonization and Co-colonization conditions, respectively. Our finding revealed that the frequency of *S. aureus* in oral-only and nasal-only swab samples was 14.1%. Moreover, oral-only and nasal-only colonization by MRSA was found in 8.2%, and 4.1% of patients, respectively. Among all patients, both oral and nasal colonization by *S. aureus* was found in 5.3% of patients.

Kearney et al. have surveyed the prevalence of MRSA isolates among patients in an acute hospital. The results of their study were similar to our findings. They showed that 6.4% of patients were colonized by MRSA in both oral and nasal(Kearney et al. 2020). In another study, Lin et al. revealed that the nasal carriage rate of S. aureus among diabetic patients with and without foot ulcers was 15.2% and 16.9%, respectively. Moreover, the results of their study showed that the nasal carriage rate of MRSA among these patients was 5.4% and 1.7%, respectively(Lin et al. 2020). The nasal colonization rate of *S. aureus* in studies performed by Baroja et al. from Ecuador(Baroja et al. 2021), Chen et al. from Taiwan(Chen et al. 2020), Wu et al. from Taiwan(Wu et al. 2017), and Bassetti et al. from Switzerland(Bassetti et al. 2005) were higher than our study.

These studies found that the nasal carriage of *S. aureus* in patients was 23.7%, 26.3%, 31.7%, and 34.6%, respectively.

In contrast, the oral colonization rate of *S. aureus* in studies carried out by Wu et al. from Taiwan(Wu et al. 2017), Bassetti et al. from Switzerland(Bassetti et al. 2005), and Silva et al. from Portugal(Simoes-Silva et al. 2018) was 18.8%, 34.6%, and 90.5%, respectively. Moreover, the nasal colonization rate of MRSA in studies performed by Baroja et al.(Baroja et al. 2021), Chen et al.(Chen et al. 2020), Ning et al.(Ning et al. 2020), and Wu et al.(Wu et al. 2017) was 5%, 17.5%, 23.4%, and 4.4%, respectively.

In Iran, some studies have surveyed the frequency of *S. aureus* in cancer patients. The frequency of *S. aureus* in studies performed by Kalantar et al. in 2014 and Montazeri et al. in 2021 was 5% and 12.4%, respectively(Kalantar et al. 2014; Abbasi Montazeri et al. 2021).

In general, the vestibulum nasi (or anterior nares) is considered a reservoir for the spread of *S. aureus*. Nasal carriage is the main step in the pathogenesis of *S. aureus*. This pathogen uses different cell surface components and many proteins to create solid interaction with nasal epithelial cells(Kearney et al. 2020). *S. aureus* nasal colonization increases the risk of infection by 2 to 10 times and is the main risk factor for the development of community or healthcare-acquired staphylococcal infection. On the other hand, it is revealed that MRSA nasal colonization is linked to an increased risk of symptomatic and severe infections(Sakr et al. 2018). Therefore, control and prevention of *S. aureus* and MRSA nasal and oral colonization in patients, especially in immunocompromised individuals is necessary.

In the present study, we observed that the use of ciprofloxacin before hospitalization was linked to an increase in a subject’s likelihood of being colonized with *S. aureus* and MRSA. Therefore, drug self-administration was a key risk factor for *S. aureus* and MRSA colonization in our study. It is predicted that 7–52% of patients with cancer on chemotherapy and or radiotherapy have oral candidiasis(Hamzavi et al. 2019).

In the current study, oral and nasal colonization by *Candida* spp. was found in 21.2% and 2.9% of patients, respectively. Our finding revealed that *C. albicans* with 16.5% frequency had the highest prevalence among *Candida* species. Moreover, *Candida* species had the highest frequency among patients who have a history of ICU admission (oral: 29.9% versus 15.5%, nasal: 4.5% versus 1.9%). According to the antifungals used, the frequency of *Candida* colonization varies between different studies(Hamzavi et al. 2019).

Different studies have surveyed the oral colonization rate of *Candida* spp. in patients with malignancy. In studies performed by Tarapan et al. from Thailand(Tarapan, Matangkasombut et al. 2019), Kheirollahi et al. from Iran(Kheirollahi et al. 2019), Gammelsrud et al. from Norway(Gammelsrud et al. 2011), Gravina et al. from Venezuela(González Gravina et al. 2007), Jayachandran et al. from India(Jayachandran et al. 2016), and Wu et al. from Taiwan(Wu et al. 2017), the oral colonization rate of *Candida* spp. was higher than our finding. In these studies, the prevalence of *Candida* spp. in oral swab samples was 87.5%, 62.6%, 59%, 69.3%, 88.3%, and 50.6%, respectively. In contrast, the oral colonization rate of *Candida* spp. in a study performed by Hamzavi et al. from Iran was lower than in our study. Results of their study revealed that 11% of patients with hematological malignancy were positive for *Candida* spp. in oral samples(Hamzavi et al. 2019). In general, *C. albicans* had the highest frequency in all mentioned studies. Similar to our study, Hamzehee et al. in Iran identified the colonization rate of *Candida* spp. in oral mucosa in patients with hematological malignancies undergoing chemotherapy. They showed that 28% of patients had positive oral candidiasis(Hamzehee et al. 2019).

Patients on chemotherapy are the susceptible group to fungal infection. In patients with different malignancies, the oral cavity is a significantly appropriate place for the growth of microorganisms and oropharyngeal candidiasis is the main fungal infection in these subjects(Bilgic and Sozer 2017; Kheirollahi et al. 2019). Therefore, the prevention and control of invasive fungal diseases in patients suffering from cancer, especially among children is necessary. Recently, the main strategies for prevention and management of candida infections are as follows: (1) use of antifungal prophylaxis such as triazoles or pneumocandins, (2) decontamination of the upper respiratory tract, and (3) decrease of the colonization of the orointestinal tract(Safdar and Armstrong 2002; González Gravina et al. 2007; Gammelsrud et al. 2011).

The oral and nasal co-colonization of *S. aureus* and *Candida* spp. was detected in 5.9% and 0.6% of patients, respectively. Moreover, the oral co-colonization of MRSA and *Candida* spp. was detected in 4.7% of patients. Globally, limited studies have investigated the oral or nasal co-colonization rate of *S. aureus* or MRSA and *Candida* spp. Wu et al. revealed that co-colonization of *Candida* with *S. aureus* and MRSA was detected in 9.2% and 1.5% of patients, respectively(Wu et al. 2017). In another study performed by Martins et al., 7.14% of patients showed *C. albicans* and *S. aureus* in the oral rinses(Martins, Koga-Ito and Jorge 2002). *Candida* spp. and *S. aureus* have a synergistic interaction with each other. In co-colonization conditions, it is revealed that *Candida* activates the *S. aureus* proliferation and increases the virulence of this pathogen(Hu et al. 2021). This is a two-way relationship. *S. aureus* increases the antifungal resistance and pathogenicity of *Candida* and significantly increases the mortality rate among patients(Eichelberger and Cassat 2021).

In general, *mecA* is the main mechanism of methicillin resistance among *S. aureus* isolates(Kean et al. 2017). Our quantitative analysis indicated that the *mecA* and *Als3p* gene expression increased six and two times in co-colonization conditions compared to single colonization conditions, respectively. Based on our findings, *Candida* can augment the resistance of *S. aureus* to methicillin and perhaps to other clinically important antibiotics such as vancomycin. *Als3*p is the main factor in the binding of *S. aureus* to the *Candida* hyphae and is required for the transport of *S. aureus* from the tongues to the lymph nodes(Schlecht et al. 2015). Moreover, the binding of *S. aureus* to the *Candida* hyphae increases phagocytosis of this pathogen and can lead to its dissemination(Schulte et al. 2015).

The limitations of the present study are as follows: (1) In the current study, we did not have a control group to investigate and compare the *Candida* spp. and *S. aureus* colonization rate and other variables. (2) The present study was a Ph.D. thesis with a limited budget and we were unable to survey the presence and prevalence of resistance genes in *Candida* spp. and *S. aureus* isolates. (3) Our study also lacked access to patient information such as severe infectious complications or treatment outcomes, and mortality rates. 4. According to the lack of access to the data about the International Society of Paediatric Oncology (SIOP) care level of Mofid children’s hospital and annual changes in hospital policies, we were unable to perform specific analyses.

In conclusion, our findings revealed the importance of polymicrobial infection in clinical settings and stated that *Candida* spp. facilitates the infection of *S. aureus* and can lead to systemic infection in co-colonized patients. Moreover, this study showed that the nasal colonization rate of *S. aureus*, MRSA, and *Candida* spp. in the patients undergoing chemotherapy was high. Understanding the synergistic interactions between these microorganisms improves our knowledge of polymicrobial infections and highlights the importance of developing effective treatment strategies in healthcare settings.

## Data Availability

All data generated or analysed during this study are included in this published article.
